# Trastuzumab administration during pregnancy: an update

**DOI:** 10.1186/s12885-021-08162-3

**Published:** 2021-04-26

**Authors:** Angeliki Andrikopoulou, Kleoniki Apostolidou, Spyridoula Chatzinikolaou, Garyfalia Bletsa, Eleni Zografos, Meletios-Athanasios Dimopoulos, Flora Zagouri

**Affiliations:** 1 Department of Clinical Therapeutics, Alexandra Hospital, Medical School, Athens, 11528 Greece; 2 Medical School, National and Kapodistrian University of Athens, Athens, Greece; 3 Hellenic Anticancer Institute, Athens, Greece

**Keywords:** Breast cancer, Pregnancy, Gestation, Trastuzumab, her2, Oligohydramnios

## Abstract

**Background:**

Over than one third (28–58%) of pregnancy-associated breast cancer (PABC) cases are characterized by positive epidermal growth factor receptor 2-positive (HER2) expression. Trastuzumab anti-HER2 monoclonal antibody is still the benchmark treatment of HER2-positive breast tumors. However, FDA has categorized Trastuzumab as a category D drug for pregnant patients with breast cancer. This systemic review aims to synthesize all currently available data of trastuzumab administration during pregnancy and provide an updated view of the effect of trastuzumab on fetal and maternal outcome.

**Methods:**

Eligible articles were identified by a search of MEDLINE bibliographic database and ClinicalTrials.gov for the period up to 01/09/2020; The algorithm consisted of a predefined combination of the words “breast”, “cancer”, “trastuzumab” and “pregnancy”. This study was performed in accordance with the PRISMA guidelines.

**Results:**

A total of 28 eligible studies were identified (30 patients, 32 fetuses). In more than half of cases, trastuzumab was administered in the metastatic setting. The mean duration of trastuzumab administration during gestation was 15.7 weeks (SD: 10.8; median: 17.5; range: 1–32). Oligohydramnios or anhydramnios was the most common (58.1%) adverse event reported in all cases. There was a statistically significant decrease in oligohydramnios/anhydramnios incidence in patients receiving trastuzumab only during the first trimester (*P* = 0.026, Fisher’s exact test). In 43.3% of cases a completely healthy neonate was born. 41.7% of fetuses exposed to trastuzumab during the second and/or third trimester were born completely healthy versus 75.0% of fetuses exposed exclusively in the first trimester. All mothers were alive at a median follow-up of 47.0 months (ranging between 9 and 100 months). Of note, there were three cases (10%) of cardiotoxicity and decreased ejection fraction during pregnancy.

**Conclusions:**

Overall, treatment with trastuzumab should be postponed until after delivery, otherwise pregnancy should be closely monitored.

## Background

Pregnancy-associated breast cancer (PABC) is defined as any breast carcinoma diagnosed during pregnancy or during the first postpartum year [1]. It occurs in 1 to 3000 pregnancies while it has been estimated that up to 3% of breast cancers may be diagnosed in pregnant women [1, 2]. The incidence of PABC is also increasing due to advanced maternal age in today’s society. Median age of disease is 33 years (23–47 years), while there is a 2- to 3-fold decreased risk of PABC in women younger than 30 [1]. Interestingly enough, there is an increased incidence (54–80%) of estrogen receptor (ER) – negative tumors in pregnancy-related tumors [1]. This could be explained by downregulation of the receptors as a negative feedback effect of estrogen and progesterone upon hormonal receptor expression. The greater incidence of ER-negative breast cancer in pregnant women mainly stems from the young age of onset. However, some studies demonstrated that the percentage of ER-positive pregnancy-associated breast cancers was not significantly different from that of non-pregnant age-matched patients [3, 4]. On the other hand, epidermal growth factor receptor 2 -positive (HER2) tumors compose the 28–58% of PABC [3–5]. Although *Elledge* et al. found 7 out of 12 pregnant patients (58%) to be positive for HER2, *Middleton* et al. found no difference in the HER2 expression rate (28%) between pregnant and young nonpregnant women [3, 4]. *Amant* et al. reported an 31.8% incidence of HER2-positive tumors in pregnant women which is consistent with the results provided by *Cardonick* et al (27%) [6, 7]. Overall, the incidence of HER2-positive tumors was approximately equal to this of patients with breast cancer younger than 35 years old (39%), although it still remains a significant proportion [8].

Treatment of pregnant women with breast cancer represents a clinically challenging case in terms of maternal and fetal safety. Treatment of HER-2 positive PABC relies on the administration of trastuzumab anti-HER2 monoclonal antibody which remains the standard-of-care for all HER2-positive breast tumors. Trastuzumab binds HER2 on the C-terminal portion of domain IV and inhibits HER2 proteolytic cleavage and release of the extracellular domain in breast cancer cells [9]. Cells treated with trastuzumab undergo arrest during the G1 phase of the cell cycle leading to reduced proliferation. Trastuzumab exerts its antitumor activity through antibody-dependent cell-mediated cytotoxicity. However, our knowledge remains limited on the use and safety of trastuzumab during pregnancy because of its cytotoxic nature. Adverse effects of trastuzumab treatment include hematological and gastrointestinal disorders as well as cardiovascular effects that could potentially threaten pregnancy outcome.

In vivo studies conducted in cynomolgus monkeys at doses up to 25 times that of the weekly human maintenance dose of 2 mg/kg Herceptin revealed no evidence of harm to the fetus. However, trastuzumab transfer through the placenta has been observed during the early (days 20–50 of gestation) and late (days 120–150 of gestation) pregnancy period [10]. A warning about trastuzumab administration during pregnancy states that administration should be avoided during gestation unless it is mandatory for mother’s health. As for patients with breast cancer that become pregnant while receiving Trastuzumab or within 7 months after the last dose, close monitoring is indispensable.

The aim of this systematic review is to provide un updated consensus regarding trastuzumab administration during pregnancy after synthesizing all existing data emerging from case reports and individual cases. We previously conducted a relevant systematic review assessing exposure to trastuzumab during pregnancy that was published in 2012 [11]. Since there is new emerging evidence from additional cases during all these years, an updated review of literature would contribute to revision of existing data and reconsideration of current practice.

## Methods

This systematic review was performed in accordance with PRISMA guidelines [12]. Eligible articles were identified by a search of MEDLINE bibliographic database and ClinicalTrials.gov for the period up to September 2020. The search algorithm consisted of the following keywords: (breast AND (carcinoma OR carcinomas OR cancer OR cancers OR neoplasm OR neoplasms)) AND (pregnancy OR pregnant OR gestation) AND (trastuzumab OR herceptin). In order to maximize the amount of synthesized information, we meticulously examined the reference lists of the relevant reviews and articles retrieved for potentially eligible papers. Language restrictions were not applied. All studies that examined the efficacy and safety of trastuzumab during pregnancy were eligible for this systematic review, no matter of sample size. All cases where therapeutic or spontaneous abortion occurred were excluded. In addition, articles assessing trastuzumab administration before or after the gestation period were considered ineligible. Eligible studies required the administration of trastuzumab at some point during pregnancy even if treatment commenced prior to pregnancy initiation. Moreover, reviews were ineligible, while all prospective and retrospective studies, as well as case reports, were eligible for this systematic review. In cases where overlapping publications emerging from the same study were identified, the larger size study was included. Two independently working reviewers (FZ and AA) performed the selection of studies and any disagreements were resolved by team consensus.

Data extraction comprised the following: general information (first author’s name, study year, journal, title), patient age at pregnancy, patient age at breast cancer diagnosis, histopathological diagnosis, clinical stage at times of disease and pregnancy diagnosis, treatment regimens administered during pregnancy, gestational age (GA) at trastuzumab initiation and withdrawal, gestational age at delivery, way of delivery and birth weight, adverse effects of chemotherapy during pregnancy, fetal and mother outcome. The quantitative synthesis of the all the recruited articles was divided in two parts. First, the descriptive statistics regarding the age of breast cancer patients at pregnancy and at BC diagnosis, GA at delivery, GA at breast cancer diagnosis, GA at trastuzumab administration, stage of disease, duration of trastuzumab administration during pregnancy, birth weight of the neonate and way of delivery were calculated. Second, the association between the occurrence of oligohydramnios/anhydramnios and the following parameters was examined: (1) exposure to trastuzumab during the second/third trimester (vs. exclusive exposure during the first trimester), (2) duration of trastuzumab administration (in weeks). Statistical analysis was performed with SPSS 24.0 statistical software.

## Results

**Fig. 1:**
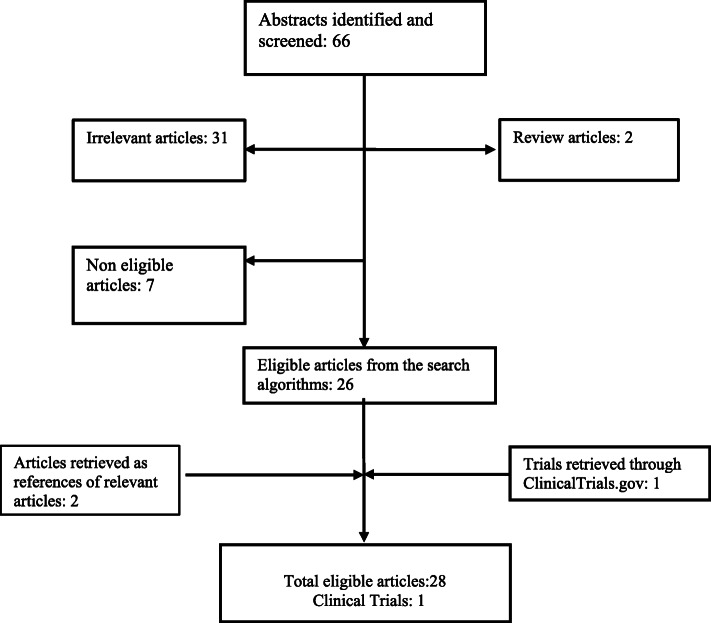
Flowchart presenting the successive steps during the selection of studies

Figure 1 presents the successive steps of the selection of eligible studies. Overall, the search algorithm recruited 66 articles. Two articles were reviews examining trastuzumab administration during pregnancy [11, 13], while 31 articles were deemed irrelevant. There were 5 additional cases where the patient declined chemotherapy during pregnancy and thus treatment with trastuzumab was withhold until after delivery [14–18]. These articles were not eligible for our study. In a case report by *Berveiller* et al trastuzumab treatment was not administered during gestation and thus was excluded [19]. One article by *Azim H.A.* et al reported all pregnancy events in patients enrolled in HERA trial during or after exposure to trastuzumab [20]. There is no detailed information regarding each one case and therefore the study was not included in our analysis. However, this important study is discussed extensively in the discussion section. Two additional articles were retrieved from the thorough search of the reference lists of eligible articles [21, 22]. From the three clinical trials identified in ClinicalTrials.gov only one study was considered eligible (MOTHER trial), although results are not yet published [23]. Taken as a whole, 28 articles were finally included in our systematic analysis (Table 1).

**Table 1:** All eligible studies and case reports of trastuzumab administration during pregnancy in breast cancer patients

Author	Treatment during pregnancy	Pathological type, Grade	Stage at Pregnancy	Age at BC diagnosis	Age at pregnancy	GA at trastuzumab	GA at delivery	Delivery	Fetal outcome	AEs during pregnnacy	Initial Staging	PFS	OS
Yildirim et al. 2018 [9]	Trastuzumab, Pertuzumab	IDC, ER: -, PR: -, HER2: +	IV (liver, lung bone)	22	23	Prior to pregnancy - 20th GA week	Not delivered	–	Elective abortion at 27th GA week	Oligohydramnios/Anhydramnios, Right renal agenesis, IUGR, Right adrenal gland hyperplasia	IV	NR	NR
Rasenack et al. 2016 [10]	Trastuzumab	IDC, ER: +, PR: +, HER2: +	IV (retroperitoneal, supraclavicular, mediastinal, left hilar, upper abdominal LNs)	25	29	Prior to pregnancy – 24th GA week, 29th GA week	35th + 5 week	Cesarean section	Healthy at 3 years old, 2735 g birth weight, Apgar 7/9/9	Oligohydramnios at 24th week, Recovered after trastuzumab interruption, Reappeared at 29th week after 8th trastuzumab dose	pT2N0M0 (08/2004)	> 72 months	> 72 months
Safadi et al. 2012 [11]	Trastuzumab, Vinorelbine	IDC scirrhous, ER: -, PR: -, HER2: +, Gr3	IV (bone)	32	32	30th GA week	33th + 5 week	Cesarean section	Healthy at 13 months, 1990 g birth weight, Apgar 8/9/9	Anhydramnios at 33 weeks	IV	> 13	> 13
Mandrawa et al. 2011 [12]	Trastuzumab	IDC, ER: -, PR: -, HER2: +,	IV (brain)	25	28	Prior to pregnancy - 27th GA week (9 doses in total, 3510 mg)	37 weeks	Vaginal delivery	Healthy at 28 months, 3060 g. Birth weight, Transient Tachypnoea of the newborn	Oligohydramnios at 25th week, recovered after 2 weeks, recurred in 3d trimester	TxN0M0	2,75	> 52,25 months
Roberts et al. 2010 [13]	Trastuzumab	IDC, ER: -, PR: -, HER2: +, Gr3	T2N1M0	36	36	4th GA week to 21st GA week	37 weeks	Vaginal delivery	Healthy, 3200 g birth weight, Mild Transient Tachypnoea of the Newborn and CPAP for 24 h	Cardiotoxicity (LVEF decline: 61 to 40%, CHF)	T2N1M0	> 9,25	> 9,25
Beale et al. 2009 [15]	Trastuzumab, Tamoxifen	IDC, ER: +, HER2: +, Gr3	TxNxM0	28	29	Prior to pregnancy - 22nd week, already received 9 doses of trastuzumab	31 + 6 weeks	Cesarean section	**Twin A**: 1590 g, Apgar 5/8/9, Intubated at 8 min for respiratory failure, Chronic renal failure and chronic lung disease, Death due to respiratory distress at 3 months **Twin B**: Healthy at discharge, 1705 g, Apgar 8/10 Transient respiratory failure till day 3, Elevated creatinine	Severe oligohydramnios, recovered in Twin B but remained minimal in Twin A, Amnioinfusion in 30 + 2′ weeks, Premature rupture of membranes (PROM)	TxNxM0	> 14	> 14
Smith et Warraich 2009 [16]	Trastuzumab, Tamoxifen, Goserelin	IDC, ER: +, HER2: +, Gr3	TxNxM0	35	35	7th GA week - 31st week	37 weeks	Cesarean section	Severe pulmonary hypoplasia and atelectasis, 2690 g birth weight, Death at 40 min after extubation	Persistent anhydramnios from 28th GA week till delivery	TxNxM0	> 14.25	> 14.25
Pant et al. 2008 [17]	Trastuzumab	IDC, Gr2/3, ER: -, PR: -, HER2: +	IV (lung)	30	32	Prior to pregnancy -30th week, total dose 4200 mg	32 + 1 weeks	Vaginal delivery	Healthy at 5 years old, Normal Apgar values, 1810 g birth weight	Oligohydramnios from 25 to 32d week, premature rupture of membranes (PROM)	IIA (T1N1M0)- Radical mastectomy & Lymph node dissection (2 years before)	NR	> 129.5
Witzel et al. 2008 [18]	Trastuzumab	IDC, ER: +, PR: -, HER2: +, Gr2.	IV (lung, brain)	29	31	Prior to pregnancy -27th GA weeks (9 cycles in total, total dose 56 mg/kg)	27 weeks	Cesarean section	Severe respiratory distress and strong capillary leak syndrome, necrotizing enterocolitis, 1015 g birth weight, Apgar 8/7/6, Death due to multiple organ failure at 5 months	Oligohydramnios and severe vaginal bleeding at 27th GA week,	T2NxM0 After neoadjuvant: pT0N0M0	> 1	> 37.25
Sekar and Stone 2007 [19]	Trastuzumab, Docetaxel	IDC, ER: -, PR: -, HER2: +, Gr2	IV (lung, brachial plexus)	25	28	23^d^ GA week - 27th GA week (docetaxel 380 mg total dose, 1385 mg trastuzumab total dose)	36 + 2 weeks	Cesarean section	Healthy at delivery, 2230 g birth weight, Apgar 7/9	Anhydramnios and IUGR at 30th GA week	T2N2M0 (Radical mastectomy & Lymphadenectomy	> 22	> 100
Waterston and Graham (2006) [20]	Trastuzumab	IDC, Gr2, ER: -, PR: -, HER2: +	II (TxN1M0)	30	30	Prior to pregnancy – 3^d^ GA week, total dose 523 mg during pregnancy	Term	Vaginal delivery	Healthy at delivery	No complications	II (TxN1M0)	> 9.25	> 9.25
Fanale et al. 2005 [21]	Trastuzumab, Vinorelbine	IDC, Gr3, ER: -, PR: -, HER2: +	IV (liver)	26	26	27th GA week - 34th GA week	34 + 5 weeks	Vaginal delivery	Healthy at 6 months old, 2270 g birth weight, Apgar score 9/9/10	Oligohydramnios	IIB (T2N1M0)	> 3	> 18.75
Watson et al. 2005 [22]	Trastuzumab	IDC, ER: -, PR: -, HER2: +	T2N3M0	28	28	Prior to pregnancy - 20th GA week	37,5 weeks	Vaginal delivery	Healthy at 6 months old, 2960 g birth weight, Apgar score 8/9	Anhydramnios	T2N3M0	> 16.5	> 16.5
Berwart et al. (2020) [23]	Trastuzumab, Tamoxifen	Left: IDC, ER: +, PR: +, HER2: + Right: IDC, ER: +, PR: +, HER2: -	T2N0M0	31	32	Prior to pregnancy - 16th GA week Docetaxel: 20th GA week – 32^d^ GA week	38 weeks	Cesarean section	Healthy at 3 years old, 3820 g birth weight	No complications	T2N1M0 (Left mastectomy + Lymphadenectomy)	12	> 48
Safi et al. (2019) [24]	Trastuzumab, Docetaxel, Cyclophosphamide	NR	NR	NR	NR	3d trimester	36 weeks	Vaginal Delivery	Mild Respiratory distress, 2380 g birth weight, Apgar score 10, Admitted to Special Care Nursery (SCN) and discharged on day 4	No complications	NR	NR	NR
Aktoz et al. 2020 [25]	Trastuzumab, Docetaxel	IDC, ER: -, PR: -, HER2: +	IV (liver)	37	37	22nd - 34th GA week (5 cycles)	35 + 3 weeks	Cesarean section	Healthy at delivery, 2850 g birth weight, Apgar 8/8/9	No complications	IV (liver)	> 3.5	> 3.5
Lambertini et al. 2019 [26]	Patient 3: Trastuzumab, Brain RT Patient 4: Trastuzumab, Lapatinib, Tamoxifen (**12 patients**)	NR	NR	NR	Median:33 (30.0–36.5)	**Patient 1,2**: Prior to pregnancy – 3 months prior to pregnancy **Patient 3,4**: 1st trimester	Patient 3: 34 weeeks Median: 39 (36.5–39.5)	Patient 3: Cesarean section 3 Cesarean sections/ 1 vaginal delivery/ 1 missing	7/12 (58.3%) Elective abortion No spontaneous abortions Median birth weight 3145 g (2880–3776) Apgar 8–9/9–10	Patient1,2: No complications Patient 3: IUGR Patient 4: No complications No oligohydramnios No congenital malformations	NR	Patient 3: 1 Patient 4: -	Patient 3: 2 Patient 4: -
Shlensky et al. 2017 [27]	Trastuzumab, Doxorubicin, Cyclophosphamide, Paclitaxel	IDC, ER: -, PR: -, HER2: +	IV	NR	NR	15th GA week	33	Vaginal delivery	Healthy, Normal birth weight, 5 min Apgar score > 7	Oligohydramnios at 33d GA week	IV	NR	NR
Andrade et al. 2016 [28]	Trastuzumab	IDC, ER: -, PR: -, HER2: +, Gr2	III (T3N2M0)	31	32	Prior to pregnancy – 27th GA week and then 28th -31st GA week (11 cycles in total, 4400 mg total dose)	32 + 2 weeks	Cesarean section	Respiratory distress syndrome/Pulmonary infection, 1655 g birth weight, Apgar 4/10, Pulmonary hypertension/Persistence of the arterial canal Low creatinine clearance (6.1 ml/min), Healthy at 7 years old	Oligohydramnios at 27th GA week, Anhydramnios at 31st GA week	III (T3N2M0)	32	> 96
Pianca et al. 2015 [29]	Trastuzumab	IDC, ER: -, PR: -, HER2: +, Gr2	T2N0M0	30	31	2d trimester – 28th GA week (2 cycles in total)	37th week	Cesarean section	2735 g birth weight, Apgar 4/8, O2 therapy at delivery, Healthy at 7 years old	Small abdominal circumference, Oligohydramnios at 29th GA week	T2N0M0	> 11.75	> 11.75
Gottschalk et al. 2011 [30]	Trastuzumab, Docetaxel, Carboplatin	IDC, ER: +, PR: +, HER2: +, Gr2 + DCIS	TxNxM0	38	38	14th GA week – 20th GA week weekly (7 cycles, 4 mg/kg)	33 + 2 weeks	Cesarean section	Dystrophic premature neonate at delivery, birth weight < 3rd percentile, Postpartum normal development and renal function	Anhydramnios, Fetal renal failure at 21st GA week, IUGR at 28th week	TxNxM0	> 5.9	> 5.9
Azim et al. 2012 [31]	Trastuzumab (16 patients)	TxNxM0/ Non metastatic	NR	NR	32.5 (26–40)	3 months prior to pregnancy – during pregnancy	40 (39–40) (*n* = 5)	NR	Healthy, Mean birth weight: 3485 (2940–4180), Mean Apgar score (10 min): 10 (9–10)	7 (44%) induced abortions 25% (4/16) spontaneous abortions No oligohydramnios No congenital abnormalities	TxNxM0	NR	NR
Goodyer et al. 2009 [32]	Trastuzumab (2 patients)	Patient 1: ER: -, PR: -, HER2: + Patient 2: ER: -, PR: -, HER2: +	Patient 1: IV (pleural effusion) Patient 2: III	Patient 1: 30 Patient 2: 36	NR	Patient 1: Second trimester – 29th GA week Patient 2: Prior to pregnancy – 6th GA week	Patient 1: 29 weeks Patient 2: 39 weeks	Patient 1: Cesarean section Patient 2: Vaginal Delivery	**Patient 1:** Respiratory distress syndrome and conductive hearing loss at delivery, Mild hypertonia and hyperreflexia, 1220 g birth weight, Healthy at 3 years old with ongoing minimal tightness of Achilles tendon **Patient 2:** Healthy at 2 years old, 2940 g birth weight, Events of gastroenteritis at 3, 8, 11 months	Patient 1: - Patient 2: 1 of 2 viable fetal sacs	Patient 1: TxN + M0 Patient 2: III	Patient 1: > 2 Patient2: > 24	Patient 1: >` 36 Patient2: > 24
Azim et al. 2009 [33]	Trastuzumab	IDC, ER: -, PR: -, HER2: +, Gr3	II (T2N1M0)	29	30	Prior to pregnanacy - 1st GA week (1 cycle, 6 mg/kg)	39 weeks	Cesarean section	Healthy at 14 moths old, 3550 g birth weight,	No complications	II (T2N1M0)	> 46	> 46
Schoendorfer et Schaefer 2008 [34]	Trastuzumab	NR	IV (lung)	NR	32	Prior to pregnancy – 23^d^ GA week	27 + 4 weeks	Cesarean section	Multiple prematurity-related problems, Dysplastic/hypoplastic left kidney and renal congestion, Death at 4 months	Oligohydramnios at 23^d^ GA week, Premature detachment of the placenta at 28th GA week	IV (lung)	> 8.25	> 8.25
Shrim et al. 2007 [35]	Trastuzumab	IDC, ER: -, PR: -, HER2: +, Gr3	IV (lung, brain)	28	32	Prior to pregnancy – 24th GA week (3200 mg total dose)	37 weeks	Cesarean section	Healthy at 2 months old, 2600 g birth weight, Apgar 9/10, Transient tachypnea of the newborn, No maternal HF	Decreased maternal LVEF at 18th and 24th GA weeks	TxNxM0	> 22	> 100
Berveiller et al. 2008 [36]	Trastuzumab	ER: -, PR: -, HER2: +	III (T2N2bM0)	43	45	Prior to pregnancy (14 months, 2 mg/kg)	–	–	Voluntary abortion	Cervico-isthmic pregnancy	III (T2N2bM0)	> 23	> 23
Bader et al. 2007 [37]	Trastuzumab, Paclitaxel	ER: -, PR: +, HER2: +	IV (bone mets, spinal cord compression)	31	38	25th – 28th GA week (2 cycles, 14 mg/kg total dose)	32 + 1 weeks	Cesarean section	Bacterial sepsis, transient renal failure, RDS at delivery, 1460 g birth weight, Healthy at 3 months	Anhydramnios and IUGR at 32d GA week	I	> 7.75	> 16.75
Diakite et al. 2019 [38]	Trastuzumab	IDC, Gr2, ER: -, PR: +, HER2: +	T4N2aMx	32	33	Prior to pregnancy –first trimester	33d GA week	Cesarean section	**Twin A:** Respiratory distress, 1450 g birth weight, Death at 10 days **Twin B**: 1550 g birth weight, Death at 40 days due to cardiorespiratory arrest	Fetal distress and oligohydramnios	T1NxMx	> 19.25	> 19.25
Gupta et al. 2014 [39]	Trastuzumab, Paclitaxel (Dexamethazone, RT)	IDC, Gr3, ER: -, PR: -, HER2: +	IV (brain)	24	24	Prior to pregnancy – 12th GA week & 3^d^ trimester – 6 weeks postpartum	38 weeks	Cesarean section	Apgar 9/9, Healthy at 6 months old Maternal LVEF mildly decreased, Disease progression in brain mets/Leptomeningeal spread Death at 6 months postpartum	Brain metastases at 22nd GA week No fetal complications	T4N3cMx	2.5	23

Overall, 30 patients and 32 fetuses were exposed to trastuzumab during pregnancy [21, 22, 24–49] Table 1. Trastuzumab was administered during pregnancy as a monotherapy regimen in most cases [22, 25, 27, 28, 31, 32, 34, 36, 39, 43, 45–48] or in combination with pertuzumab [24], vinorelbine [26, 35], paclitaxel [21, 49], docetaxel [33, 41], docetaxel and carboplatin [44], tamoxifen [29, 30, 37], tamoxifen and lapatinib [42], docetaxel and cyclophosphamide [40], doxorubicin and cyclophosphamide and paclitaxel [38] and also concurrently with brain RT in one case [42]. The mean age of patients at pregnancy was 31.1 years (SD: 3.96; median: 31.5; range 23–38) [21, 22, 24–37, 39, 41, 43, 44, 46–49], while the mean age at breast cancer diagnosis was 29.9 years (SD: 4.21; median: 30.0; range: 22–38) [20–22, 24–37, 39, 41, 43–46, 48, 49]. In more than half of cases, trastuzumab was administered in the metastatic setting [21, 24–27, 31–33, 35, 38, 39, 41, 42, 45, 47–49], while in the remaining cases it was administered in the adjuvant setting [22, 28–30, 34–37, 39, 43–46].

Evaluating available histologies, invasive ductal carcinoma (IDC) was diagnosed in all known cases [21, 22, 24–27, 29–39, 41, 43, 44, 46, 48], while in one case invasive lobular carcinoma (ILC) co-existed [32]*.* The tumor was estrogen receptor (ER) - positive in 23% of the cases [25, 29, 30, 32, 37, 44] and progesterone receptor (PR) - positive in 20,8% of the cases [22, 25, 37, 44, 49]. Breast cancer was human epidermal growth factor receptor 2 (HER2) – positive in all included cases [21, 22, 24–49].

The mean duration of trastuzumab administration during gestation was 15.7 weeks (SD: 10.8; median: 17.5; range: 1–32) [24–39, 41, 43–49]. Overall, 23.3% of patients were exposed to trastuzumab during all trimesters of pregnancy [25, 27, 30–32, 39, 43]. Importantly, 20.0% of patients with breast cancer were exposed to trastuzumab exclusively during the first trimester [22, 34, 42, 45, 46] while trastuzumab was also administered during the second or third trimester in 80.0% of the cases [21, 24–33, 35–41, 43–45, 47–49].

Oligohydramnios or anhydramnios was the most common (58.1%) adverse event reported in all cases [22, 24–27, 29–33, 35, 36, 38, 39, 43, 44, 47, 49]. Only one of the six cases (16.7%) of trastuzumab exposure exclusively during the first trimester of gestation was complicated with oligohydramnios or anhydramnios. In contrast, seventeen out of 24 pregnancies (70.8%) where trastuzumab was administered during the second or/and third trimester were complicated with oligohydramnios or anhydramnios. The difference was statistically significant (*P* = 0.026, Fisher’s exact test). The trend pointing to a positive association between the duration of trastuzumab treatment and the development of oligohydramnios or anhydramnios did not reach statistical significance (OR = 1.05, 95% CI: 0.96–1.14, increment: 1 week, *P* = 0.316).

In 67.9% of cases, delivery was performed via a cesarean section [21, 22, 25, 26, 29, 30, 32, 33, 37, 39, 41–49], while in nine pregnancies (32.1%) there a vaginal delivery occurred [27, 28, 31, 34–36, 38, 40, 45]. The mean gestational age at delivery was 34.6 weeks (SD: 3.26; median: 35.4; range: 27–39) [22, 25–33, 35–49], whereas the mean birth weight at delivery was 2371 g (SD: 771.2; median: 2490; range: 1015–3820) [22, 25–28, 30–33, 35–37, 39–41, 43, 45, 46, 48, 49].

In thirteen cases (43.3%), a completely healthy neonate (thirteen out of 32 neonates) was born [21, 25, 26, 31, 33–38, 41, 45, 46]. In the remaining cases, neonates presented with: renal agenesis/hypoplasia (2 cases) [24, 47], mild transient tachypnoea (three cases) [27, 28, 48], respiratory distress syndrome (six cases) [22, 39, 40, 43, 45, 49], respiratory failure (two cases) [29, 32], renal failure (three cases) [29, 47, 49], transient respiratory failure and elevated creatinine (one case) [29], severe pulmonary hypoplasia (one case) [30], capillary leak syndrome and necrotizing enterocolitis (one case) [32], pulmonary hypertension and persistence of arterial canal (one case) [39], prematurity-related disorders (two cases) [44, 47], conductive hearing loss and mild hypertonia/hyperreflexia (one case) [45], bacterial sepsis (one case) [49] and cardiorespiratory arrest (one case) [22].

Of note, 41.7% (10 out of 24) of fetuses exposed to trastuzumab during the second and/or third trimester were born completely healthy [21, 25, 26, 31, 33, 35–38, 41] in contrast with 75.0% of fetuses exposed exclusively in the first trimester [34, 45, 46]. However, the sizeable numerical statistical significance was not achieved (*P* = 0.311; Fisher’s exact test). Once again, the trend pointing to a negative association between the duration of trastuzumab administration and the delivery of a completely healthy neonate did not reach statistical significance (OR = 0.921, 95% CI: 0.85–1.00, *P* = 0.061).

As far as maternal outcome is concerned, all patients with breast cancer were alive at a median follow-up of 47.0 months (ranging between 9 and 100 months), while only two patients relapsed during follow-up according to existing data. It should be noted that there were three cases (10%) of cardiotoxicity and decreased ejection fraction during pregnancy [21, 28, 48].

Detailed information of all eligible studies is provided in Table 1.

## Discussion

PABC is a rare but complex entity which demands multidisciplinary management. *Amant* et al. reported similar survival rates between pregnant patients with breast cancer and the matched non-pregnant population, despite the preceding belief that PABC is associated with a poor outcome [6]. The finding that survival rates of pregnant BC patients are comparable to those of nonpregnant is essential for mother counselling and optimization of PABC management. Breast cancer treatment during pregnancy does not jeopardize maternal prognosis.

Specific guidelines have been developed for PABC treatment. Surgery is not contraindicated during pregnancy and the type of surgery chosen should be based on usual criteria (mastectomy versus breast conserving surgery) [50]. The lack of wound complications in pregnant BC patients supports surgical management of PABC. Despite the concern of milk fistulae development and that of postoperative hematoma due to the hypervascularization of the breasts, there was no apparent increase in surgical complications between pregnant and nonpregnant patients [50].

Chemotherapy should be started after the first trimester and should be stopped 2–3 weeks prior to delivery for a chemotherapy-free interval. The teratogenicity of chemotherapy depends on time of exposure, dose administered and placental transfer. During the first 2 weeks of pregnancy, spontaneous abortion is more common after chemotherapy treatment, rather than teratogenic effects on the fetus. However, as organogenesis happens from 2nd to 8th gestational week, the embryo is more vulnerable to congenital malformations during this period. The risk of chemotherapy-induced congenital malformations during the 1st trimester is 20%, whereas it declines to 1–2% during the 2nd and 3d trimester [51–53].

Regarding trastuzumab administration during pregnancy, we report that in approximately two third of cases oligohydramnios or anhydramnios were developed. The risk for intrauterine complications and oligohydramnios/anhydramnios development was minimal in pregnancies were fetal exposure to trastuzumab occurred exclusively during the first trimester (16.7% vs 70.8%; *P* = 0.026). In addition, a completely healthy neonate was born in 75% of cases that trastuzumab treatment affected only the first trimester in contrast with 41.7% of cases where the fetus was exposed during second and/or third trimester as well, although this association failed to reach statistical significance (*P* = 0.311; Fisher’s exact test). Our results are consistent with existing knowledge. Fetal exposure to trastuzumab is considered to be low during the first trimester while it gradually increases during the second half of gestation to reach mother levels at delivery [54]. Trastuzumab in an IgG1 monoclonal antibody with a molecular mass that does not permit transport across the placenta via simple diffusion. Active transport of these antibodies require binding to the Fc receptor of the syncytiotrophoblast, however Fc receptor is hardly detectable before the 14th week of gestation. Therefore, placental transfer of trastuzumab during the first trimester is minimal [54]. Antibody transfer to fetal endocrine organs has been reported from 4th to 6th week of development, although concentration was rather low [55]. Indeed, fetuses exposed to trastuzumab exclusively in the 1st trimester tend to be healthy at delivery. This observation is important for the correct consultation of women diagnosed with pregnancy while being on trastuzumab treatment.

US FDA has categorized Trastuzumab as a category D drug due to fetal complications [56]. No congenital malformations or teratogenic effects were reported in our review, apart from some prematurity-related problems [44, 47]. Indeed, animal studies did not reveal any teratogenic effects even at doses up to 25 times the recommended weekly human dose [56].

The most common fetal complication observed in our study was oligohydramnios or anhydramnios during second or third trimester. This is a result of EGFR receptor blockade in fetal renal epithelium by trastuzumab, where EGFR receptors are highly expressed [57]. Indeed, EGFR binding affinity in human fetal kidney between 6 and 11th GA week is 4–5 times greater than in normal renal tissue. This abundance of EGFR binding sites in fetal kidney falls rapidly in the postnatal period to low levels observed in adult renal epithelium [57]. EGF increases DNA synthesis in human fetal kidney cells, while anti-EGFR antibody leads to the opposite result. Therefore, trastuzumab attenuates the important role of EGFR receptors in fetal renal cell proliferation and nephrogenesis resulting in the aforementioned cases of oligo−/anhydramnios, fetal renal failure [29, 47, 49] and renal agenesis [24, 47] reported in our study. In addition, even in the presence of normal kidneys, this receptor blockade leads to decreased urinary output and empty fetal bladder visualization. Another explanation of the trastuzumab-induced oligohydramnios is the decreased expression of vascular endothelial growth factor (VEGF). VEGF regulates amniotic fluid production and absorption via modulation of the rate of intramembranous absorption of amniotic fluid by both passive and nonpassive mechanisms [58]. Trastuzumab downregulates VEGF expression and may affect the amniotic fluid level.

Another possible mechanism of trastuzumab-mediated reduction of amniotic fluid may be through altering the function of aquaporins, a family of cell membrane water channels responsible for intramembranous fluid exchange in various tissues as proposed by Sekar and Stone [33]. More specifically, Aquaporin-3 is expressed in placenta, chorion, and amnion and regulation of its expression may contribute to amniotic fluid homeostasis [59]. Moreover, it was shown that Aquaporin-8 and Aquaporin-9 levels were significantly decreased in amnion and increased in placenta in fetuses suffering from oligohydramnios, further supporting the effect of aquaporins in amniotic fluid levels [60].

Whatever the mechanism, there is increased evidence that oligohydramnios induced by trastuzumab is reversible upon discontinuation of treatment [25, 27, 29]. The mean half-life elimination time for trastuzumab weekly schedule is 6 days (range: 1–32 days) and for the 3-week schedule 16 days (range: 11–23 days) [53]. This may indicate the time required for recovery of oligo−/anhydramnios after trastuzumab treatment. Moreover, it has been shown that the risk of oligo/anhydramnios is analogous to the duration of exposure to trastuzumab during pregnancy. Trastuzumab exposure for a relatively short period does not seem to substantially affect the pregnancy outcome. In contrast, a more prolonged period of exposure is associated with increased risk of fetal harm. In our study, there was a trend to a positive association between the duration of trastuzumab treatment and the development of oligohydramnios or anhydramnios, although not statistically significant (OR = 1.05, 95% CI: 0.96–1.14, increment: 1 week, *P* = 0.316).

Berveiller et al. reported one case of ectopic cervico-isthmic pregnancy while on trastuzumab treatment [19]. ErbB2 is required embryo implantation process [61]. On days 1–4 ErbB2 mRNA is expressed in uterine epithelial cells, while on days 6–8 the mRNA was accumulated in both implantation and interimplantation sites [61]. Given the crucial role of HER2 in embryo implantation process, it could be postulated that trastuzumab was responsible for the incidence of this ectopic pregnancy.

*Azim* et al explored the effect of previous or concurrent trastuzumab administration on pregnancy outcome based on data emerging from HERA trial, one of the largest Phase III trials evaluating trastuzumab treatment in the adjuvant setting [20]. Azim et al. reported that 25% of pregnancies that occurred while on trastuzumab treatment resulted in spontaneous abortions. In consistence with our results, no congenital anomalies were reported in the study. However, there were also no cases of oligohydramnios or anhydramnios recorded in the study. The study demonstrated that women that conceived after a period of 3 months after trastuzumab cessation had an uneventful pregnancy, a finding that contributes significantly to the existing knowledge [20]. Of note, results from the very interesting MOTHER trial are anticipated [23].

## Conclusions

Overall, medical oncologists encounter the dilemma of choosing between the optimal therapy for the mother and survival of the fetus. Considering that trastuzumab is equally effective when administered within 6 months from breast cancer diagnosis, it may be delayed until after delivery [62]. However, if trastuzumab administration is inevitable as in the case of metastatic disease, close monitoring of both mother and the fetus is required. It should be noted that inadvertent conception while taking Herceptin during the first trimester only is not an indication for termination.

*NR: Not reported.*

## Data Availability

Data supporting our findings can be found in PubMed bibliographical database and ClinicalTrials.gov website. Links providing these data are listed below: https://pubmed.ncbi.nlm.nih.gov/25853260/ https://www.wjpmr.com/home/article_abstract/1874 https://pubmed.ncbi.nlm.nih.gov/30110018/ https://www.thieme-connect.com/products/ejournals/abstract/10.1055/s-0035-1559647 https://pubmed.ncbi.nlm.nih.gov/22381111/ https://pubmed.ncbi.nlm.nih.gov/21806575/ https://www.ncbi.nlm.nih.gov/pmc/articles/PMC2853413/ https://pubmed.ncbi.nlm.nih.gov/19398090/ https://pubmed.ncbi.nlm.nih.gov/19274553/ https://pubmed.ncbi.nlm.nih.gov/18349415/ https://europepmc.org/article/med/18084047 https://pubmed.ncbi.nlm.nih.gov/17666645/ https://pubmed.ncbi.nlm.nih.gov/16401684/ https://pubmed.ncbi.nlm.nih.gov/16277887/ https://pubmed.ncbi.nlm.nih.gov/15738038/ https://pubmed.ncbi.nlm.nih.gov/32729389/ https://pubmed.ncbi.nlm.nih.gov/31488880/ https://pubmed.ncbi.nlm.nih.gov/30153764/ https://pubmed.ncbi.nlm.nih.gov/30335191/ https://pubmed.ncbi.nlm.nih.gov/28255521/ https://pubmed.ncbi.nlm.nih.gov/26825868/ https://www.ncbi.nlm.nih.gov/pmc/articles/PMC5624665/ https://www.ncbi.nlm.nih.gov/pmc/articles/PMC3290009/ https://casesjournal.biomedcentral.com/articles/10.1186/1757-1626-2-9329 https://europepmc.org/article/med/19483741 https://pubmed.ncbi.nlm.nih.gov/18396008/ https://europepmc.org/article/med/17399946 https://pubmed.ncbi.nlm.nih.gov/17196514/

## Abbreviations

PABC: Pregnancy-associated breast cancer
HER2: Human epidermal growth factor receptor 2
FDA: Food and drug administration
PRISMA: Preferred reporting items for systematic reviews and meta-analyses
GA: Gestational age
BC: Breast cancer
AE: Adverse event
IDC: Invasive ductal carcinoma
ER: Estrogen receptor
PR: Progesterone receptor
ILC: invasive lobular carcinoma
DCIS: Ductal carcinoma in Situ
LNs: Lymph nodes
Gr: Grade
LVEF: *Left ventricular ejection fraction*
CHF: Congestive heart failure
EGFR: *Epidermal growth factor receptor*
ErbB2: Erb-B2 receptor tyrosine kinase 2
VEGF: Vascular endothelial growth factor
RT: Radiation therapy
IUGR: Intrauterine growth restriction
RDS: Respiratory distress syndrome
PROM: Premature rupture of membranes
CPAP: Continuous positive airway pressure
SCN: Special care nursery
IgG1: Immunoglobulin G1
CI: Confidence interval
*OR*: Odds ratio
*SD*: Standard deviation
NR: Not reported
