# Correction to: Trastuzumab administration during pregnancy: an update

**DOI:** 10.1186/s12885-021-09087-7

**Published:** 2021-12-17

**Authors:** Angeliki Andrikopoulou, Kleoniki Apostolidou, Spyridoula Chatzinikolaou, Garyfalia Bletsa, Eleni Zografos, Meletios-Athanasios Dimopoulos, Flora Zagouri

**Affiliations:** 1 Department of Clinical Therapeutics, Alexandra Hospital, Medical School, 11, ,528 Athens, Greece; 2 Medical School, National and Kapodistrian University of Athens, Athens, Greece; 3 Hellenic Anticancer Institute, Athens, Greece

**Correction to: BMC Cancer 21, 463 (2021)**

**https://doi.org/10.1186/s12885-021-08162-3**

Following publication of the original article [1], the authors identified reference citation errors in Table 1. The corrected Table 1 is supplied below.

**Table 1:** All eligible studies and case reports of trastuzumab administration during pregnancy in breast cancer patients

Author	Treatment during pregnancy	Pathological type, Grade	Stage at Pregnancy	Age at BC diagnosis	Age at pregnancy	GA at trastuzumab	GA at delivery	Delivery	Fetal outcome	AEs during pregnnacy	Initial Staging	PFS	OS
Yildirim et al. 2018 (24)	Trastuzumab, Pertuzumab	IDC, ER: -, PR: -, HER2: +	IV (liver, lung bone)	22	23	Prior to pregnancy - 20th GA week	Not delivered	–	Elective abortion at 27th GA week	Oligohydramnios/Anhydramnios, Right renal agenesis, IUGR, Right adrenal gland hyperplasia	IV	NR	NR
Rasenack et al. 2016 (25)	Trastuzumab	IDC, ER: +, PR: +, HER2: +	IV (retroperitoneal, supraclavicular, mediastinal, left hilar, upper abdominal LNs)	25	29	Prior to pregnancy – 24th GA week, 29th GA week	35th + 5 week	Cesarean section	Healthy at 3 years old, 2735 g birth weight, Apgar 7/9/9	Oligohydramnios at 24th week, Recovered after trastuzumab interruption, Reappeared at 29th week after 8th trastuzumab dose	pT2N0M0 (08/2004)	> 72 months	> 72 months
Safadi et al. 2012 (26)	Trastuzumab, Vinorelbine	IDC scirrhous, ER: -, PR: -, HER2: +, Gr3	IV (bone)	32	32	30th GA week	33th + 5 week	Cesarean section	Healthy at 13 months, 1990 g birth weight, Apgar 8/9/9	Anhydramnios at 33 weeks	IV	> 13	> 13
Mandrawa et al. 2011 (27)	Trastuzumab	IDC, ER: -, PR: -, HER2: +,	IV (brain)	25	28	Prior to pregnancy - 27th GA week (9 doses in total, 3510 mg)	37 weeks	Vaginal delivery	Healthy at 28 months, 3060 g. Birth weight, Transient Tachypnoea of the newborn	Oligohydramnios at 25th week, recovered after 2 weeks, recurred in 3d trimester	TxN0M0	2,75	> 52,25 months
Roberts et al. 2010 (28)	Trastuzumab	IDC, ER: -, PR: -, HER2: +, Gr3	T2N1M0	36	36	4th GA week to 21st GA week	37 weeks	Vaginal delivery	Healthy, 3200 g birth weight, Mild Transient Tachypnoea of the Newborn and CPAP for 24 h	Cardiotoxicity (LVEF decline: 61 to 40%, CHF)	T2N1M0	> 9,25	> 9,25
Beale et al. 2009 (29)	Trastuzumab, Tamoxifen	IDC, ER: +, HER2: +, Gr3	TxNxM0	28	29	Prior to pregnancy - 22nd week, already received 9 doses of trastuzumab	31 + 6 weeks	Cesarean section	**Twin A**: 1590 g, Apgar 5/8/9, Intubated at 8 min for respiratory failure, Chronic renal failure and chronic lung disease, Death due to respiratory distress at 3 months **Twin B**: Healthy at discharge, 1705 g, Apgar 8/10 Transient respiratory failure till day 3, Elevated creatinine	Severe oligohydramnios, recovered in Twin B but remained minimal in Twin A, Amnioinfusion in 30 + 2′ weeks, Premature rupture of membranes (PROM)	TxNxM0	> 14	> 14
Smith et Warraich 2009 (30)	Trastuzumab, Tamoxifen, Goserelin	IDC, ER: +, HER2: +, Gr3	TxNxM0	35	35	7th GA week - 31st week	37 weeks	Cesarean section	Severe pulmonary hypoplasia and atelectasis, 2690 g birth weight, Death at 40 min after extubation	Persistent anhydramnios from 28th GA week till delivery	TxNxM0	> 14.25	> 14.25
Pant et al. 2008 (31)	Trastuzumab	IDC, Gr2/3, ER: -, PR: -, HER2: +	IV (lung)	30	32	Prior to pregnancy -30th week, total dose 4200 mg	32 + 1 weeks	Vaginal delivery	Healthy at 5 years old, Normal Apgar values, 1810 g birth weight	Oligohydramnios from 25 to 32d week, premature rupture of membranes (PROM)	IIA (T1N1M0)- Radical mastectomy & Lymph node dissection (2 years before)	NR	> 129.5
Witzel et al. 2008 (32)	Trastuzumab	IDC, ER: +, PR: -, HER2: +, Gr2.	IV (lung, brain)	29	31	Prior to pregnancy -27th GA weeks (9 cycles in total, total dose 56 mg/kg)	27 weeks	Cesarean section	Severe respiratory distress and strong capillary leak syndrome, necrotizing enterocolitis, 1015 g birth weight, Apgar 8/7/6, Death due to multiple organ failure at 5 months	Oligohydramnios and severe vaginal bleeding at 27th GA week,	T2NxM0 After neoadjuvant: pT0N0M0	> 1	> 37.25
Sekar and Stone 2007 (33)	Trastuzumab, Docetaxel	IDC, ER: -, PR: -, HER2: +, Gr2	IV (lung, brachial plexus)	25	28	23^d^ GA week - 27th GA week (docetaxel 380 mg total dose, 1385 mg trastuzumab total dose)	36 + 2 weeks	Cesarean section	Healthy at delivery, 2230 g birth weight, Apgar 7/9	Anhydramnios and IUGR at 30th GA week	T2N2M0 (Radical mastectomy & Lymphadenectomy	> 22	> 100
Waterston and Graham (2006) (34)	Trastuzumab	IDC, Gr2, ER: -, PR: -, HER2: +	II (TxN1M0)	30	30	Prior to pregnancy – 3^d^ GA week, total dose 523 mg during pregnancy	Term	Vaginal delivery	Healthy at delivery	No complications	II (TxN1M0)	> 9.25	> 9.25
Fanale et al. 2005 (35)	Trastuzumab, Vinorelbine	IDC, Gr3, ER: -, PR: -, HER2: +	IV (liver)	26	26	27th GA week - 34th GA week	34 + 5 weeks	Vaginal delivery	Healthy at 6 months old, 2270 g birth weight, Apgar score 9/9/10	Oligohydramnios	IIB (T2N1M0)	> 3	> 18.75
Watson et al. 2005 (36)	Trastuzumab	IDC, ER: -, PR: -, HER2: +	T2N3M0	28	28	Prior to pregnancy - 20th GA week	37,5 weeks	Vaginal delivery	Healthy at 6 months old, 2960 g birth weight, Apgar score 8/9	Anhydramnios	T2N3M0	> 16.5	> 16.5
Berwart et al. (2020) (37)	Trastuzumab, Tamoxifen	Left: IDC, ER: +, PR: +, HER2: + Right: IDC, ER: +, PR: +, HER2: -	T2N0M0	31	32	Prior to pregnancy - 16th GA week Docetaxel: 20th GA week – 32^d^ GA week	38 weeks	Cesarean section	Healthy at 3 years old, 3820 g birth weight	No complications	T2N1M0 (Left mastectomy + Lymphadenectomy)	12	> 48
Safi et al. (2019) (40)	Trastuzumab, Docetaxel, Cyclophosphamide	NR	NR	NR	NR	3d trimester	36 weeks	Vaginal Delivery	Mild Respiratory distress, 2380 g birth weight, Apgar score 10, Admitted to Special Care Nursery (SCN) and discharged on day 4	No complications	NR	NR	NR
Aktoz et al. 2020 (41)	Trastuzumab, Docetaxel	IDC, ER: -, PR: -, HER2: +	IV (liver)	37	37	22nd - 34th GA week (5 cycles)	35 + 3 weeks	Cesarean section	Healthy at delivery, 2850 g birth weight, Apgar 8/8/9	No complications	IV (liver)	> 3.5	> 3.5
Lambertini et al. 2019 (42)	Patient 3: Trastuzumab, Brain RT Patient 4: Trastuzumab, Lapatinib, Tamoxifen (**12 patients**)	NR	NR	NR	Median:33 (30.0–36.5)	**Patient 1,2**: Prior to pregnancy – 3 months prior to pregnancy **Patient 3,4**: 1st trimester	Patient 3: 34 weeeks Median: 39 (36.5–39.5)	Patient 3: Cesarean section 3 Cesarean sections/ 1 vaginal delivery/ 1 missing	7/12 (58.3%) Elective abortion No spontaneous abortions Median birth weight 3145 g (2880–3776) Apgar 8–9/9–10	Patient1,2: No complications Patient 3: IUGR Patient 4: No complications No oligohydramnios No congenital malformations	NR	Patient 3: 1 Patient 4: -	Patient 3: 2 Patient 4: -
Shlensky et al. 2017 (38)	Trastuzumab, Doxorubicin, Cyclophosphamide, Paclitaxel	IDC, ER: -, PR: -, HER2: +	IV	NR	NR	15th GA week	33	Vaginal delivery	Healthy, Normal birth weight, 5 min Apgar score > 7	Oligohydramnios at 33d GA week	IV	NR	NR
Andrade et al. 2016 (39)	Trastuzumab	IDC, ER: -, PR: -, HER2: +, Gr2	III (T3N2M0)	31	32	Prior to pregnancy – 27th GA week and then 28th -31st GA week (11 cycles in total, 4400 mg total dose)	32 + 2 weeks	Cesarean section	Respiratory distress syndrome/Pulmonary infection, 1655 g birth weight, Apgar 4/10, Pulmonary hypertension/Persistence of the arterial canal Low creatinine clearance (6.1 ml/min), Healthy at 7 years old	Oligohydramnios at 27th GA week, Anhydramnios at 31st GA week	III (T3N2M0)	32	> 96
Pianca et al. 2015 (43)	Trastuzumab	IDC, ER: -, PR: -, HER2: +, Gr2	T2N0M0	30	31	2d trimester – 28th GA week (2 cycles in total)	37th week	Cesarean section	2735 g birth weight, Apgar 4/8, O2 therapy at delivery, Healthy at 7 years old	Small abdominal circumference, Oligohydramnios at 29th GA week	T2N0M0	> 11.75	> 11.75
Gottschalk et al. 2011 (44)	Trastuzumab, Docetaxel, Carboplatin	IDC, ER: +, PR: +, HER2: +, Gr2 + DCIS	TxNxM0	38	38	14th GA week – 20th GA week weekly (7 cycles, 4 mg/kg)	33 + 2 weeks	Cesarean section	Dystrophic premature neonate at delivery, birth weight < 3rd percentile, Postpartum normal development and renal function	Anhydramnios, Fetal renal failure at 21st GA week, IUGR at 28th week	TxNxM0	> 5.9	> 5.9
Azim et al. 2012 (20)	Trastuzumab (16 patients)	TxNxM0/ Non metastatic	NR	NR	32.5 (26–40)	3 months prior to pregnancy – during pregnancy	40 (39–40) (*n* = 5)	NR	Healthy, Mean birth weight: 3485 (2940–4180), Mean Apgar score (10 min): 10 (9–10)	7 (44%) induced abortions 25% (4/16) spontaneous abortions No oligohydramnios No congenital abnormalities	TxNxM0	NR	NR
Goodyer et al. 2009 (45)	Trastuzumab (2 patients)	Patient 1: ER: -, PR: -, HER2: + Patient 2: ER: -, PR: -, HER2: +	Patient 1: IV (pleural effusion) Patient 2: III	Patient 1: 30 Patient 2: 36	NR	Patient 1: Second trimester – 29th GA week Patient 2: Prior to pregnancy – 6th GA week	Patient 1: 29 weeks Patient 2: 39 weeks	Patient 1: Cesarean section Patient 2: Vaginal Delivery	**Patient 1:** Respiratory distress syndrome and conductive hearing loss at delivery, Mild hypertonia and hyperreflexia, 1220 g birth weight, Healthy at 3 years old with ongoing minimal tightness of Achilles tendon **Patient 2:** Healthy at 2 years old, 2940 g birth weight, Events of gastroenteritis at 3, 8, 11 months	Patient 1: - Patient 2: 1 of 2 viable fetal sacs	Patient 1: TxN + M0 Patient 2: III	Patient 1: > 2 Patient2: > 24	Patient 1: > 36 Patient2: > 24
Azim et al. 2009 (46)	Trastuzumab	IDC, ER: -, PR: -, HER2: +, Gr3	II (T2N1M0)	29	30	Prior to pregnanacy - 1st GA week (1 cycle, 6 mg/kg)	39 weeks	Cesarean section	Healthy at 14 moths old, 3550 g birth weight,	No complications	II (T2N1M0)	> 46	> 46
Schoendorfer et Schaefer 2008 (47)	Trastuzumab	NR	IV (lung)	NR	32	Prior to pregnancy – 23^d^ GA week	27 + 4 weeks	Cesarean section	Multiple prematurity-related problems, Dysplastic/hypoplastic left kidney and renal congestion, Death at 4 months	Oligohydramnios at 23^d^ GA week, Premature detachment of the placenta at 28th GA week	IV (lung)	> 8.25	> 8.25
Shrim et al. 2007 (48)	Trastuzumab	IDC, ER: -, PR: -, HER2: +, Gr3	IV (lung, brain)	28	32	Prior to pregnancy – 24th GA week (3200 mg total dose)	37 weeks	Cesarean section	Healthy at 2 months old, 2600 g birth weight, Apgar 9/10, Transient tachypnea of the newborn, No maternal HF	Decreased maternal LVEF at 18th and 24th GA weeks	TxNxM0	> 22	> 100
Berveiller et al. 2008 (19)	Trastuzumab	ER: -, PR: -, HER2: +	III (T2N2bM0)	43	45	Prior to pregnancy (14 months, 2 mg/kg)	–	–	Voluntary abortion	Cervico-isthmic pregnancy	III (T2N2bM0)	> 23	> 23
Bader et al. 2007 (49)	Trastuzumab, Paclitaxel	ER: -, PR: +, HER2: +	IV (bone mets, spinal cord compression)	31	38	25th – 28th GA week (2 cycles, 14 mg/kg total dose)	32 + 1 weeks	Cesarean section	Bacterial sepsis, transient renal failure, RDS at delivery, 1460 g birth weight, Healthy at 3 months	Anhydramnios and IUGR at 32d GA week	I	> 7.75	> 16.75
Diakite et al. 2019 (22)	Trastuzumab	IDC, Gr2, ER: -, PR: +, HER2: +	T4N2aMx	32	33	Prior to pregnancy –first trimester	33d GA week	Cesarean section	**Twin A:** Respiratory distress, 1450 g birth weight, Death at 10 days **Twin B**: 1550 g birth weight, Death at 40 days due to cardiorespiratory arrest	Fetal distress and oligohydramnios	T1NxMx	> 19.25	> 19.25
Gupta et al. 2014 (21)	Trastuzumab, Paclitaxel (Dexamethazone, RT)	IDC, Gr3, ER: -, PR: -, HER2: +	IV (brain)	24	24	Prior to pregnancy – 12th GA week & 3^d^ trimester – 6 weeks postpartum	38 weeks	Cesarean section	Apgar 9/9, Healthy at 6 months old Maternal LVEF mildly decreased, Disease progression in brain mets/Leptomeningeal spread Death at 6 months postpartum	Brain metastases at 22nd GA week No fetal complications	T4N3cMx	2.5	23
